# “Innovative diagnostics and treatments of infections in transplantation,” report from the 2025 *spring highlights in transplantation sciences* meeting

**DOI:** 10.3389/ti.2026.16562

**Published:** 2026-08-10

**Authors:** Jacques Fourgeaud, Benedicte Neven, Nassim Kamar, Imane Farhat, Oriol Manuel, Martina Sester, Lionel Couzi, David Boutboul, Sophie Caillat Zucman, Jean Daniel Lelievre, Marie-Benedicte Le Stang, Marion Le Maréchal, Hélène François, Antoine Durrbach, Julien Zuber, Hannah Kaminski

**Affiliations:** 1 Université Paris Cité, Fédération pour la recherche en explorations et thérapeutiques innovantes in utero (FETUS), Paris, France; 2 Microbiology Department, Assistance Publique Hopitaux de Paris (AP-HP), Hôpital Necker, Paris, France; 3 Department of Pediatric Immunology, Hematology and Rheumatology, Necker-Enfants Malades Hospital, Assistance Publique Hopitaux de Paris (AP-HP), Paris, France; 4 Department of Nephrology and Organ Transplantation, Toulouse University Hospital, Institut National de la Santé et de la Recherche Médicale (INSERM) Unité Mixte de Recherche (UMR) 1291, Toulouse Institute for Infectious and Inflammatory Diseases (Infinity), Université de Toulouse, Toulouse, France; 5 Department of Nephrology, Centre Hospitalier Universitaire (CHU) Dijon, Dijon, France; 6 Service des Maladies Infectieuses et Centre de Transplantation D’organes, Centre Hospitalier Universitaire Vaudois, Lausanne, Switzerland; 7 Department of Transplant and Infection Immunology, Saarland University, Homburg, Germany; 8 Department of Nephrology, Transplantation, Dialysis and Apheresis, Bordeaux University Hospital, Bordeaux, France; 9 Hôpital Cochin, Assistance Publique-HÃ'pitaux de Paris, Université Paris Cité, Paris, France; 10 Institut National de la Santé et de la Recherche Médicale (INSERM) Unité Mixte de Recherche (UMR) 1342, Institut de Recherche Saint-Louis, Université Paris Cité, Paris, France; 11 Faculté de Médecine, Institut National de la Santé et de la Recherche Médicale (INSERM) U955, Team 16, Vaccine Research Institute, Université Paris-Est Créteil, Créteil, France; Assistance Publique-Hôpitaux de Paris, Groupe Henri-Mondor Albert-Chenevier, Service de maladies Infectieuses et Immunologie Clinique, Créteil, France; 12 Department of Kidney and Metabolic Diseases, Transplantation and Clinical Immunology, Necker Hospital, Assistance Publique Hopitaux de Paris (AP-HP), Paris, France; 13 Infectious and Tropical Diseases Unit, Grenoble Alpes University Hospital, Grenoble, France; 14 Sorbonne Université Service de Transplantation Rénale-Néphrologie, Hôpital Pitié-Salpêtrière, Assistance Publique Hopitaux de Paris (AP-HP), Institut National de la Santé et de la Recherche Médicale (INSERM) UMR_S 1155, Paris, France; 15 Institut National de la Santé et de la Recherche Médicale (INSERM) Unité Mixte de Recherche (UMR) 1356, Universite Paris Saclay, Villejuif, France

**Keywords:** BKvirus, cell-therapy, CMV, EBV, encephalitis

## Abstract

Infectious complications remain a leading cause of morbidity and mortality after solid organ transplantation, driven by profound immunosuppression, emerging pathogens, and increasing antiviral resistance. The 2025 Spring Highlights in Transplantation Sciences (HITS) meeting, held in Paris under the auspices of the Société Francophone de Transplantation and endorsed by the European Society of Organ Transplantation, brought together international experts to discuss recent advances in the diagnosis, pathogenesis, prevention, and treatment of infections in transplant recipients. This report summarizes the key scientific presentations covering innovative approaches to viral hepatitis, cytomegalovirus (CMV), Epstein–Barr virus, human herpesvirus-8, BK polyomavirus, infectious encephalitis, and vaccination strategies. Particular emphasis was placed on the growing role of metagenomic next-generation sequencing for diagnosing unexplained infections, the integration of immune monitoring into clinical decision-making, and the development of adoptive cellular therapies, including virus-specific αβ T cells and γδ T-cell–based immunotherapies for refractory CMV infection. The meeting also highlighted emerging concepts in donor-recipient immunogenetics, novel diagnostic technologies, and personalized preventive strategies. Collectively, these advances illustrate the transition toward precision medicine in transplant infectious diseases, combining cutting-edge diagnostics, immune profiling, and innovative immunotherapeutic approaches to improve the management and outcomes of solid organ transplant recipients.

## Introduction

The 2025 *Spring Highlights In Transplantation Sciences* (*HITS*) annual meeting was held in Paris on March 27-28, under the auspices of the *Société Francophone de Transplantation* and endorsed by the *European Society of Organ Transplantation*. This second edition of *Spring HITS* focused on innovative diagnostic and therapeutic approaches to infectious diseases, mainly viral-mediated diseases, in solid organ transplant recipients (SOT). We summarize in this report the presentations and discussions around the covered topics, highlighting recent knowledge gaps and technical achievements together with expert opinions. Each topic includes original/atypical clinical presentation, diagnostic and therapeutic innovations.

### Transplant hepatitis: unusual suspects

#### Metagenomic evaluation of indeterminate hepatitis in immunocompromised patients

Hepatitis in immunocompromised individuals remains a significant diagnostic challenge due to its heterogeneous etiologies and wide range of clinical severity. Standard microbiological diagnostic methods, including targeted PCR assays and microbial culture, may fail to identify the causative infectious agents. Metagenomic next-generation sequencing (mNGS), a non-targeted approach that characterizes all nucleic acids present in a clinical sample, offers a promising alternative by enabling the detection of unexpected, rare, or novel pathogens.

Jacques Fourgeaud (Virology, Paris, France) reported that in his laboratory, from 2019 to 2022, 742 samples from 523 patients were analyzed using mNGS. This approach yielded an overall diagnostic rate of 19%, which was 2.5 times higher in immunocompromised patients compared with immunocompetent individuals [1].

Several case studies have highlighted the diagnostic performance of mNGS. Notably, mNGS enabled the identification of a novel virus, human circovirus 1 (HCirV-1), a recently described circular single-stranded DNA virus belonging to the family *Circoviridae*, in patients presenting with acute hepatitis, suggesting its potential role as a previously unrecognized pathogen in immunocompromised individuals [2–4]. In another illustrative example, Aichi virus, a non-enveloped positive-sense RNA virus of the genus *Kobuvirus* (*Picornaviridae*) typically associated with self-limited acute gastroenteritis, was detected in multiple children with chronic hepatitis and primary [5]. Following its initial detection by mNGS, subsequent investigations supported the classification of both HCirV-1 and *Aichi virus* as opportunistic pathogens.

Collectively, these findings support the integration of mNGS into the diagnostic workflow, particularly as a second-line approach when conventional testing fails to yield a diagnosis. Despite inherent limitations, including higher cost, longer turnaround times, and challenges in data interpretation, mNGS offers a substantial advantage in detecting unexpected, rare, or emerging pathogens. When used as a second-line investigation, it can significantly improve the etiological diagnosis of unexplained hepatitis in immunocompromised individuals.

#### Clinical case: Spirosplasma hepatitis diagnosed by metagenomics

Imane Farhat (Kidney Transplantation, Dijon, France) presented a case highlighting the use of metagenomic sequencing to identify the cause of hepatitis of unknown origin in a kidney transplant recipient.

A 65-year-old kidney transplant recipient was admitted with isolated fever. Initial laboratory tests revealed pancytopenia and elevated CRP levels, but conventional investigations failed to detect any pathogen. A myelogram was performed due to symptoms suggestive of macrophage activation syndrome, but results were negative. Subsequently, the patient developed fulminant hepatitis. Liver biopsy showed severe acute cytolytic hepatitis with a neutrophil-rich infiltrate, suppurative hepatocytic necrosis, and hemophagocytosis. Treatment with etoposide, N-acetylcysteine and piperacillin-tazobactam was initiated. Unfortunately, the patient succumbed to hemorrhagic complications following the liver biopsy. Posthumous shotgun metagenomics (SMg) analysis of the liver tissue identified *Spiroplasma ixodetis*, a cell wall-deficient bacterium of the class *Mollicutes* previously recognized mainly as an arthropod-associated microorganism and an exceptionally rare human pathogen, as the causative agent, demonstrating the value of metagenomics in diagnosing rare or unexpected infections in immunocompromised patients [6], particularly when the identified pathogen is highly susceptible (here to doxycycline), allowing timely diagnosis and effective treatment.

#### Enteric viruses associated hepatitis

Chronic hepatopathy is a frequent complication in immunocompromised patients, particularly in those with antibody deficiencies. This condition, characterized by chronic transaminitis and/or anicteric cholestasis, is often underrecognized and may progress to portal hypertension and its associated complications. Histologically, chronic hepatopathy is marked by lobular CD8^+^ T cell infiltration, with possible granulomas and nodular regenerative hyperplasia.

Benedicte Neven (Pediatric Immuno-hematology, Paris, France), reported eported the findings of an investigation involving 50 patients with primary or secondary antibody deficiency who developed chronic hepatopathy. A strong association with chronic enteric viral infections was identified, with viruses detected in 50% of affected patients, but not in a control group with similar immunodeficiencies and no liver abnormalities. Patients with hepatopathy exhibited an expansion of activated memory CD8^+^ T cells in peripheral blood mononuclear cells, along with enhanced type I and type II interferon responses, predominantly, though not exclusively, within the memory CD8^+^ T cell compartment. This entity was termed *enteric virus-associated hepatitis* (EVAH).

The main viruses implicated were norovirus and Aichi virus. Aichi virus infection was associated with a broader clinical phenotype, including tubulo-interstitial nephritis, kidney enlargement, and progressive renal impairment in addition to liver involvement. Notably, norovirus is typically detected in stool samples, whereas Aichi virus is more frequently identified in diseased tissues and organs. The underlying pathogenic mechanisms remain to be fully elucidated and may differ between norovirus and Aichi virus. Whether EVAH contributes to liver involvement, including nodular regenerative hyperplasia, observed in long-term transplant recipients, particularly after B-cell depletion, remains to be investigated.

**FIGURE 1:**
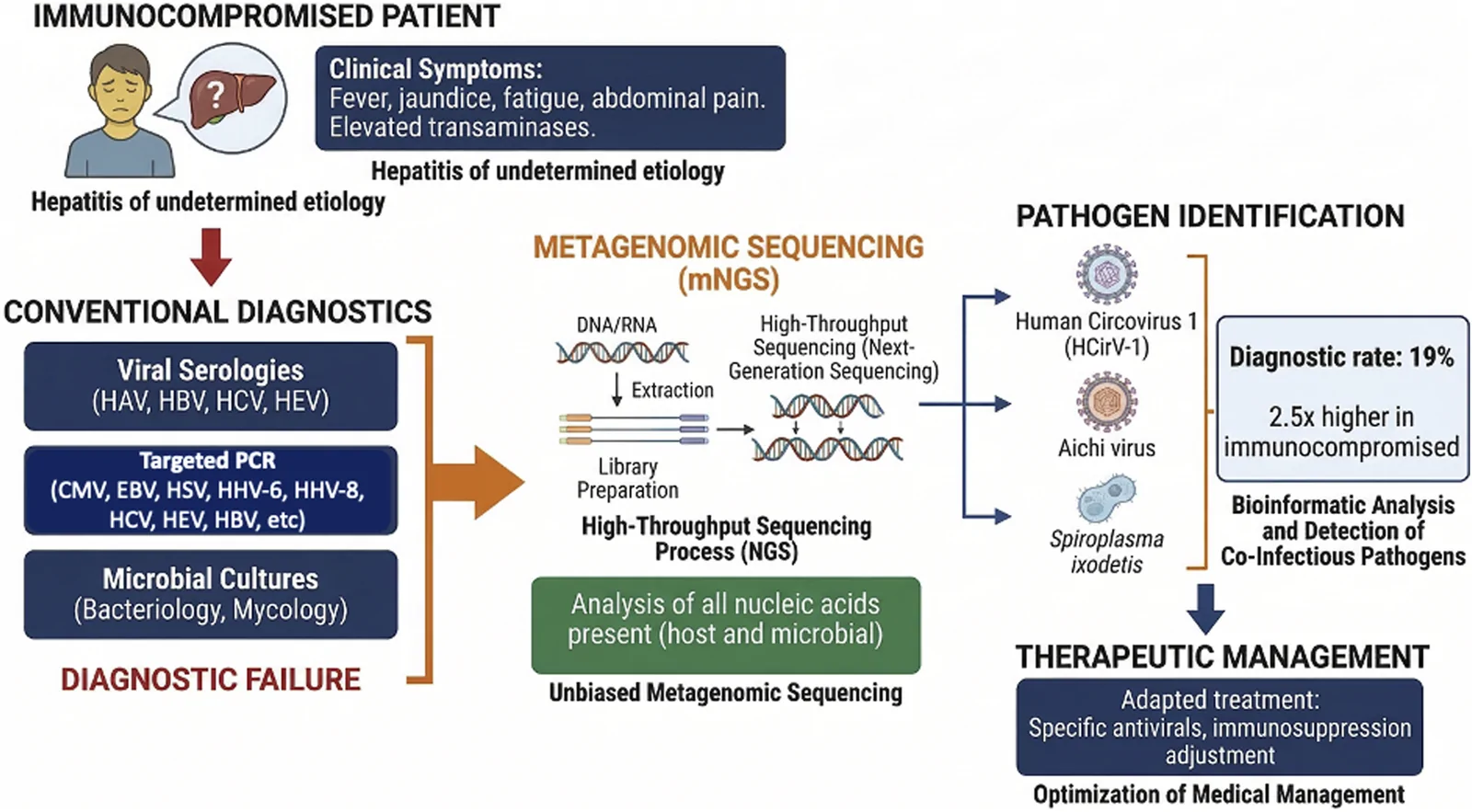
Vertical diagnostic workflow for unexplained hepatitis in immunocompromised patients. The figure highlights the role of metagenomic next-generation sequencing (mNGS) in identifying unexpected pathogens, including HCirV-1, Aichi virus, and *Spiroplasma*. Key performance metrics of mNGS are also shown.

Figure 1 highlights the role of metagenomic next-generation sequencing (mNGS) in identifying unexpected pathogens, including HCirV-1, Aichi virus, and *Spiroplasma*.

#### Viral hepatitis transmitted by the donor (B, C, E)

Nassim Kamar (Solid Organ Transplantation, Toulouse, France) provided an overview of viral hepatitis transmission through organ donation.

##### Hepatitis E (HEV)

HEV is a common cause of viral hepatitis and is often underdiagnosed in SOT recipients. It is an RNA virus with four main genotypes: genotypes 1 and 2 are human-restricted and transmitted via the fecal-oral route, whereas genotypes 3 and 4 are zoonotic. Chronic HEV infection is defined by viral replication persisting for more than 3 months. Among SOT recipients, approximately two-thirds of HEV-infected patients develop chronic hepatitis. Tacrolimus use, compared with cyclosporin A, is a major predictor of chronicity. Reducing immunosuppressive therapy may serve as a first-line intervention. Ribavirin (RBV) has demonstrated efficacy in many immunosuppressed SOT recipients, with dosing and duration tailored to viral shedding patterns. RBV discontinuation is advised only when HEV RNA is undetectable in both serum and stool. In cases of treatment failure, pegylated interferon-α may be considered in liver transplant patients but is unsafe in recipients of other organs due to the risk of rejection. Human-to-human transmission of HEV genotypes 3 and 4 is rare but possible via blood or organ transplantation. Screening is routine in the UK and France. In living donors, detection delays transplantation; for deceased donors, recipients are informed and monitored. Persistent infection beyond one to 3 months may be an indication to start RBV treatment.

##### Hepatitis C (HCV)

Current KDIGO guidelines recommend that all patients with kidney disease or undergoing transplantation who are HCV-positive receive direct-acting antiviral (DAA) therapy [7]. The timing of treatment depends on donor type, waiting list duration, liver function, and donor infection status. Since 2023 in France, organ recipients may receive transplants from anti-HCV-positive but RNA-negative donors, with PCR monitoring and DAA therapy ensuring complete viral clearance. Indeed, the risk of transmission from this group of donor is almost zero.

Conversely, HCV-positive PCR-positive donors are accepted in many other countries even for HCV-negative recipients, with good efficacy of prophylactic DAA [8].

##### Hepatitis B (HBV)

Since 2023 in France, kidneys from HBsAg-positive and/or HBV DNA-positive donors may be transplanted into recipients with chronic HBV infection, provided appropriate antiviral prophylaxis and post-transplant monitoring are maintained. Antiviral therapy is generally continued for at least 6–12 months and may be prolonged in patients with persistent HBsAg positivity or detectable HBV DNA.

In contrast, transplantation from HBV DNA-positive donors into HBV-seronegative recipients remains prohibited in France. However, this strategy has been explored in the United States in selected kidney and liver transplant recipients using intensive antiviral prophylaxis, with encouraging short-term outcomes (no chronic viremia, death or graft loss) and reduced waiting times [9].

### CMV: when a big virus turns into a big problem

#### Resistant and refractory CMV

Oriol Manuel (Transplant Infectious Diseases, Lausanne, Switzerland) discussed the incidence, therapeutic options, and challenges associated with resistant and refractory cytomegalovirus (CMV) infection in SOT recipients.

Refractory CMV remains a significant clinical challenge in this population. It is defined by an increase in or persistence of viral load despite appropriately dosed antiviral therapy, with genotypic testing confirming resistance in approximately 50% of cases [10]. While many patients eventually clear CMV without treatment modifications, high viral loads and host factors, such as donor-recipient CMV serostatus mismatch (D+/R-) or lung transplantation, increase the risk of resistance.

CMV antiviral resistance is most commonly associated with mutations in the UL97, UL54, and UL56 genes. Available therapeutic options include (val)ganciclovir, foscarnet, maribavir, CMV-specific immunoglobulins, and adoptive T-cell therapies. Maribavir, a pUL97 kinase inhibitor, offers an oral alternative to conventional therapies, with a favorable toxicity profile [11]. However, resistance to maribavir is an emerging concern, with studies showing that up to 48% of non-responders and up to 86% of patients with viral recurrence develop resistance [12]. To mitigate this risk, it is strongly recommended to initiate maribavir treatment when the viral load is below 50000UI/mL [10].

Management of refractory and resistant CMV requires a systematic approach, including adherence verification, genotypic testing, and adjustment of immunosuppressive therapy. For patients with high viral loads or end-organ disease, foscarnet remains the preferred antiviral treatment [10]. Switching to maribavir may be considered when toxicity is a concern, particularly once the viral load has been reduced [10]. The addition of CMV immunoglobulins is sometimes used in refractory cases, although robust clinical trial data are lacking. Letermovir is currently licensed for prophylaxis in D+/R- kidney transplant recipients [10]. Letermovir, used as secondary prophylaxis, may help reduce long-term maribavir exposure and prevent resistance. The combination of valganciclovir and letermovir as first-line therapy is currently being evaluated in kidney transplant recipients with CMV infection to accelerate viral clearance and reduce the risk of resistance emergence (LUCY trial; NCT06334497).

While antiviral resistance remains relatively rare, it is associated with significant morbidity, highlighting the need for ongoing research to optimize management strategies and improve outcomes for SOT recipients.

#### From a better understanding of the host-virus relationship in SOT to diagnostic and therapeutic perspectives

Martina Sester (Transplant and Infection Immunology, Homburg, Germany) presented the basic immunomodulatory strategies employed by herpesviruses, such as CMV, to evade the host immune system. These strategies include interference with innate immune mechanisms, disruption of antigen presentation, and mimicry of cytokines or other cellular proteins [13]. Recent studies have shown that mimicry between viral and host antigens not only occurs at the protein level but also at the peptide level. While short-peptide mimicry is a common feature of many viruses, herpesviruses and poxviruses more frequently exhibit mimicry of longer peptides with up to three mismatches, particularly among viruses that establish chronic infections. Viral mimicry appears to target host proteins non-randomly, notably sparing the human Y-chromosome. This suggests that viral mimicry may contribute to the development of autoimmunity while reflecting evolutionary pressure to evade the adaptive immune system and thereby establish latency [14]. These mechanisms may also help explain the unusually high levels of antigen-specific T cells required to control herpesvirus replication, including CMV [15].

Recent evidence supporting the use of CMV-specific T cells for individualized management of CMV complications in SOT recipients has been summarized in the Fourth International Consensus Guidelines on the Management of CMV in Solid Organ Transplantation of The Transplantation Society (TTS) [10]. Key recommendations include using CMV-specific T cells as an alternative to serology in patients with potential passive immunity [16–18], employing CMV-specific cellular immunity measured at day 15 post-transplant in seropositive recipients as an indicator of low risk for subsequent reactivation [19, 20], and tailoring the duration of antiviral prophylaxis (typically 3–6 months) based on the presence of CMV-specific T cells [21–24]. Furthermore, emerging data suggest that CMV-specific T cells may guide the duration of antiviral treatment, the initiation of secondary prophylaxis [25], the identification of patients at risk of relapse, and the individualized management of patients intolerant to antiviral therapy [26]. However, controlled studies are still needed to validate these applications. Overall, substantial progress in CMV immunomonitoring has now been recognized in the latest consensus guidelines, which, for the first time, include recommendations for its use in transplant recipients [10].

#### Preclinical-stage therapy: gamma delta T cells for refractory CMV disease

Lionel Couzi (Kidney Transplantation, Bordeaux, France) emphasized that, despite recent advances with maribavir, the management of refractory CMV infections remains a significant challenge in SOT recipients. There is growing interest in developing adoptive cellular therapies targeting CMV. While current HLA-restricted αβ T-cell-based therapies show promise, they have limitations, highlighting the need for complementary, HLA-independent immunotherapeutic approaches.

Human γδ T cells play a key role in the immune response against CMV [27–33], and their expansion correlates with viral recovery in SOT recipients. Notably, γδ T cells can recognize antigens via their T-cell receptor independently of MHC/HLA restriction [34] making them attractive candidates for allogeneic cell therapy. A robust, clinical-grade method has been developed for the selective, large-scale expansion of γδ T cells, referred to as “Delta One T” (DOT) cells, characterized by a predominance (>70%) of Vδ1+ cells in the final product [35]. In a preclinical study conducted by the Immunoconcept group at Bordeaux University, DOT cells were successfully expanded from both CMV-seronegative and CMV-seropositive donors. Their anti-CMV activity was demonstrated both *in vitro* and *in vivo*, using a murine “DOT-like” cell model that recapitulated the characteristics of the human product. Importantly, DOT cells were also expanded from kidney transplant patients with refractory CMV infection and retained both viability and functionality in the presence of standard immunosuppressive treatments [36].

In summary, this preclinical study provides proof-of-concept for future clinical trials evaluating autologous or allogeneic DOT cells as a novel treatment for refractory CMV disease in SOT recipients, including high-risk CMV-naïve D + R- patients.

#### CMV-specific conventional T-cell therapy

Rajiv Khanna (Biological Sciences, Brisbane, Australia) highlighted advances in CMV-specific conventional T cell therapy, which has evolved from autologous approaches in solid organ transplantation [37–40] to scalable allogeneic “off-the-shelf” products for both transplant and oncology patients [41]. Immunocompromised individuals, particularly after hematopoietic stem cell transplantation (HSCT) or solid organ transplantation (SOT), remain at high risk for severe viral diseases, including CMV, Epstein-Barr virus (EBV), BK-polyomavirus (BKPyV), and adenovirus, many of which lack effective drug treatments. Earlier work demonstrated that autologous virus-specific T cells can resolve drug-resistant viral infections and restore immunity, but manufacturing delays, limited scalability, and cost restrict their widespread use. To address this, allogeneic multi-virus-specific T cells are manufactured from carefully selected healthy donors using proprietary “pepmix” epitope pools that cover diverse HLA alleles. The Robin™ algorithm matches cryopreserved T-cell products to patients based on HLA compatibility, viral specificity, and product availability, enabling immediate treatment. Phase I clinical trials have shown that both therapeutic and prophylactic administration of these allogeneic products is safe, promotes immune reconstitution, and can lead to clearance or control of viral reactivation, even in drug-resistant cases [41]. Compassionate access programs across Australia and New Zealand have treated critically ill adults and children with various underlying diseases and following organ transplants, demonstrating encouraging clinical responses [42]. Single-cell RNA sequencing has revealed distinct functional gene signatures associated with superior survival outcomes. This platform also supports innovation in solid tumor therapy, integrating chimeric antigen receptors (CARs) targeting tumour antigens such as EphA3 [43].

Overall, this approach promises consistent, scalable, rapid-access T cell therapies with broad clinical potential for the treatment of viral infections and cancer**.**

### Navigating pathogenesis: infectious diseases challenges in transplant recipients

#### From pathophysiology to diagnostic and therapeutic perspectives for EBV and HHV-8

David Boutboul (Immuno-hematology, Paris, France), focused on Epstein-Barr virus (EBV) and human herpesvirus 8 (HHV-8), two oncogenic herpesviruses implicated in various lymphoproliferative disorders (LPDs), particularly in immunocompromised hosts.

Primary EBV infection may present with severe manifestations such as hemophagocytic lymphohistiocytosis (HLH), which can be associated with underlying genetic defects (e.g., *IL-27RA* deficiency [44]; X-linked lymphoproliferative disease, *XLP*) or arise in the context of transplantation with an donor-positive/recipient-negative (D+/R-) EBV mismatch. Chronic EBV infection is associated predominantly with B-cell LPDs and, less frequently, with T/NK-cell LPDs, which are often diagnostically challenging and may exhibit resistance to rituximab.

HHV-8, a less prevalent virus, is associated with Kaposi sarcoma (KS), the potentially life-threatening HHV-8-associated multicentric Castleman disease (MCD), and primary effusion lymphoma (PEL).

These disorders are commonly observed in the setting of secondary immunodeficiency settings, such as HIV infection or post-transplantation, but may also occur in primary immunodeficiencies, particularly as EBV-positive B-cell LPDs.

Diagnosis of EBV- and HHV-8-associated diseases relies primarily on tissue biopsy, which remains the gold standard. For EBV, Epstein-Barr virus-encoded RNA (EBER) *in situ* hybridization (ISH) co-stained with B- or T/NK-cell markers is critical, while latency-associated nuclear antigen (LANA) staining is essential for HHV-8. Whole blood viral load monitoring is also commonly used. Recently, novel diagnostic approaches, such as flowFISH (combining flow cytometry and ISH for EBER or LANA), have emerged. This technique improves the detection of infected cells, including circulating “viroblasts” in HHV-8-MCD [45], thereby facilitating rapid diagnosis and improved characterization of cellular viral reservoirs [46].

Therapeutic strategies include rituximab for EBV-associated LPDs, etoposide combined with rituximab for HHV-8-associated MCD, and immune restoration whenever feasible. Overall, this presentation highlighted the critical importance of understanding disease pathogenesis to optimize diagnostic and therapeutic strategies in these vulnerable patient populations.

#### Impact of donor HLA-DQ divergence on the control of post-transplant BKPyV infection

Sophie Caillat-Zucman (Immunology, Paris, France), presented a study examining the impact of donor and recipient HLA divergence on the risk of BK polyomavirus (BKPyV) viremia following kidney transplantation.

In the absence of specific antiviral therapies, BKPyV infection remains a major challenge after kidney transplantation, as management relies on a delicate balance between preventing allograft rejection and restoring antiviral immunity through reduction of immunosuppression [47].

The study assessed whether HLA genetic and functional diversity, by expanding the repertoire of viral peptides presented to T cells, could influence the control of post-transplant BKPyV replication in a large cohort of kidney transplant recipients. No effect of HLA diversity at any recipient locus was observed. In contrast, high genetic divergence (HED) at the donor HLA-DQ locus, reflecting heterozygosity for two highly divergent HLA-DQ heterodimer groups (i.e., DQα01 and non-DQα01), was correlated with the breadth of the BKPyV-derived immunopeptidome and independently predicted a BKPyV-free outcome.

Using a novel functional divergence metric based on the similarity of peptide-binding motifs between pairs of HLA-DQ molecules, the investigators further demonstrated that DQα01 and non-DQα01 groups are also functionally divergent. These findings provide a molecular explanation for the observed differences associated with specific HLA-DQ allele combination profiles.

Overall, the results indicate that donor HLA divergence, particularly heterozygosity for DQα01 and non-DQα01 HLA-DQ molecules, serves as a relevant proxy for the diversity of the BKPyV-derived peptides predicted to bind donor DQ molecules, and is associated with protection against BKPyV replication after kidney transplantation. These findings also underscore the critical role of DQ-restricted CD4+T cell-mediated immune responses in the control of BKPyV infection. These novel findings have recently been published [48].

#### Vaccination: physiopathology and strategies in immunocompromised patients

Jean-Daniel Lelièvre (Infectious Diseases, Créteil, France) provided an overview of vaccination strategies in immunocompromised patients.

SOT recipients are particularly susceptible to infections and often exhibit suboptimal responses to vaccination. The mechanisms underlying this impaired immunogenicity include the effects of immunosuppressive therapies, as well as immune dysfunction related to chronic organ failure, particularly chronic kidney disease. Consequently, vaccination should be initiated as early as possible, ideally prior to transplantation.

Vaccination schedules generally follow those recommended for immunocompetent individuals but often require earlier administration of certain vaccines, such as those targeting herpes zoster, pneumococcus and influenza, which are typically reserved for older adults in the general population. Strategies to enhance vaccine efficacy may include higher antigen doses, additional doses during the primary vaccination series, and more frequent booster vaccinations. Other approaches include the use of adjuvanted vaccines (e.g., influenza vaccines), heterologous vaccination schedules, and when feasible, modulation of the immunosuppressive regimen.

In addition, cocooning strategies (vaccination of close contacts) may help reduce household transmission of pathogens, including SARS-CoV-2 and *N. meningitidis*, to immunocompromised patients. Although the potential risk of graft rejection following vaccination remains a concern, a recent meta-analysis suggests that vaccination is generally safe in transplant recipients [49].

Ongoing research into novel vaccines, including those targeting norovirus and CMV, offers promising perspectives for preventing infections that are particularly relevant to immunocompromised populations. However, despite these advances, the number of clinical trials specifically involving immunocompromised patients remains limited, and evidence-based recommendations are still lacking for several vaccines.

### Targeting infectious encephalitis in transplant recipients: diagnostic and therapeutic breakthroughs

#### Pegivirus, a case series

Marie Benedicte Le Stang (Kidney Transplantation, Paris, France) reported two kidney transplant recipients who developed Pegivirus-associated encephalitis. Both patients presented with acute visual loss secondary to retrobulbar optic neuritis, accompanied by additional neurological and cognitive manifestations. Next-generation sequencing (NGS) analysis of the cerebrospinal fluid (CSF) identified pegivirus, also known as human pegivirus (HPgV) as the causative agent of encephalomyelitis. Human pegivirus (HPgV) is an enveloped positive-sense RNA virus of the family *Flaviviridae* that commonly establishes persistent asymptomatic infection in humans and has only recently been implicated in neurological disease in severely immunocompromised patients.

This virus is carried by approximately one-sixth of the population, with a higher prevalence in South Africa and Asia. Seven distinct genotypes have been described. HPgV can infect splenic and bone marrow lymphocytes and is transmitted through percutaneous exposure, blood transfusion, and sexual contact. The infection may persist in a latent state. An estimated 26% of SOT patients are thought to be infected, especially within the first year post-transplantation.

HPgV infection has also been associated with an increased risk of non-Hodgkin lymphoma and encephalomyelitis, likely through infection of microglia and astrocytes. Diagnosis relies on brain biopsy, detection of viral RNA in serum and cerebrospinal fluids (CSF), and NGS analysis of CSF. The recent report of four cases over a two-year period from a single German institution suggests that this condition may be underdiagnosed and warrants greater clinical awareness [50]. Despite a poor prognosis in immunocompromised patients, there is currently no established therapeutic consensus or effective treatment strategy.

#### Diagnosis of indeterminate encephalitis in immunocompromised patients

Marion Le Maréchal (Infectious Diseases, Grenoble, France) provided an overview of the diagnostic approach to encephalitis in immunocompromised patients. Diagnosis in this population is particularly challenging because of the broad differential diagnosis, which includes hematological malignancies, treatment-related neurotoxicity, autoimmune encephalitis, and posterior reversible encephalopathy syndrome (PRES).

The most frequent infectious causes of encephalitis in immunocompromised patients overlap with those observed in immunocompetent individuals, notably herpes simplex virus type 1 (HSV-1), varicella-zoster virus (VZV), and *Listeria monocytogenes*. However, several pathogens are more specifically associated with immunosuppression, including EBV, CMV, JC virus, and *Cryptococcus neoformans*. The spectrum of likely pathogens varies according to the type and intensity of immunosuppression, such as that seen in SOT recipients, hematopoietic stem cell transplant recipients, or patients living with HIV. Diagnostic evaluation should follow a systematic and structured approach. First-line investigations should consistently include HSV-1 and VZV polymerase chain reaction (PCR) testing and cerebrospinal fluid (CSF) cultures. In HIV-positive patients, the diagnostic work-up should additionally screen for EBV, CMV, JC virus, *Mycobacterium tuberculosis*, *C. neoformans*, *Toxoplasma gondii*, syphilis, and central nervous system lymphoma as a key differential diagnosis.

In solid organ transplant recipients, investigations should include screening for JC virus, CMV, *C. neoformans*, Mucorales*, Toxoplasma gondii*, and *Aspergillus* spp. Adjunctive tests such as serum or CSF galactomannan and β-D-glucan assays may be useful in the diagnosis of invasive fungal infections. When blood and CSF investigations remain inconclusive, early consideration of brain biopsy is warranted in immunocompromised patients. Diagnosis and therapeutic decisions should be guided by a multidisciplinary team approach.

**FIGURE 2:**
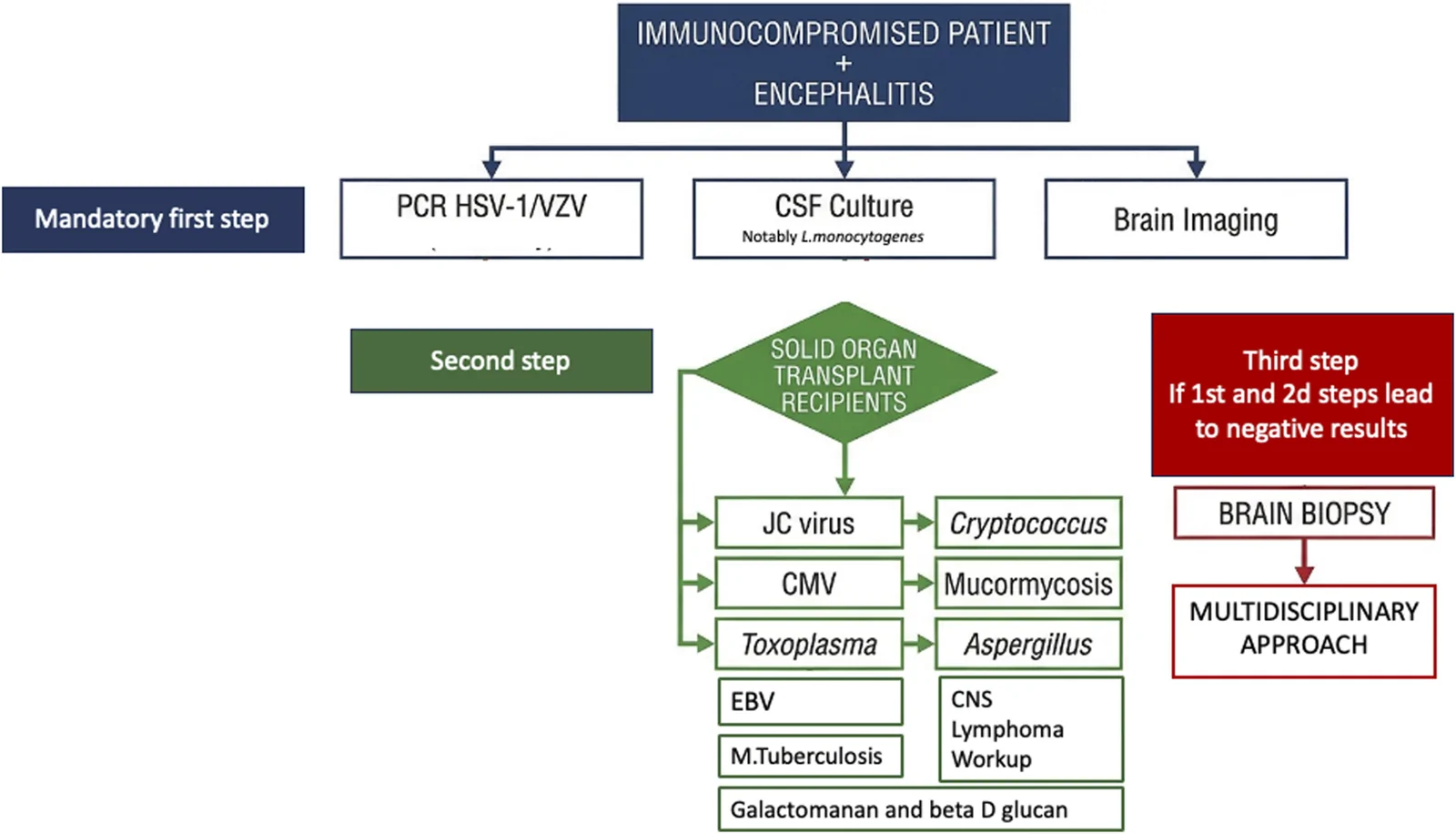
The figure illustrated the proposed diagnostic algorithm for Infectious Encephalitis for immunocompromised patients.

Figure 2 summarize the diagnostic algorithm proposed for suspected Infectious encephalis.

#### Encephalitis caused by JC virus in immunocompromised patients

Progressive multifocal leukoencephalopathy (PML) is an opportunistic infectious disease affecting the central nervous system and is caused by the JC virus. Although it has been extensively studied in patients with HIV infection or those receiving immunotherapies, data on kidney transplant recipients (KTRs) remain limited. Hélène François (Kidney Transplantation, Paris, France) reported the results of a retrospective, multicenter, nationwide study. All kidney transplant centers in France were contacted to identify cases of PML, and the total number of KTRs was obtained from the registry of the French regulatory agency.

A total of 24 cases of PML were identified. The estimated minimum nationwide incidence of PML was 4.7 per 100,000 patient-years. The median time from kidney transplantation to PML diagnosis was 88 months (interquartile range: 9–355 months), and 8 of the 24 patients (33%) had been exposed to belatacept. Almost all patients underwent a reduction or withdrawal of immunosuppression, and 13 patients (54%) received PML-specific treatment such as anti-PD1/PDL1 (15.4%) or Interleukin 7 (11.5%). Fifteen patients (63%) died within 1 year of diagnosis; these patients had greater exposure to belatacept compared with those who survived.

In this nationwide study, the incidence of PML in KTRs was at least 4.7 per 100,000 patient-years, with a 1-year mortality rate of 63%. Further investigations are warranted to confirm a potential association between belatacept exposure and the occurrence of PML. This study has recently been published [51].

## Conclusion

The Spring HITS meeting highlighted the growing complexity of infectious diseases in solid organ transplant recipients and underscored the need for integrated diagnostic and therapeutic strategies. Across a wide spectrum of viral infections, ranging from hepatitis viruses and enteric viruses to herpesviruses, polyomaviruses, and emerging neurotropic pathogens, the presentations emphasized how atypical clinical manifestations, prolonged viral persistence, and treatment resistance are tightly linked to the altered immune landscape of transplant recipients.

A major recurring theme was the transformative role of advanced diagnostics, particularly metagenomic NGS, which has proven invaluable for identifying unexpected or novel pathogens in unresolved hepatitis and encephalitis. These tools, combined with refined immunomonitoring approaches such as virus-specific T-cell assessment and HLA divergence analysis, offer new opportunities to better stratify risk, personalize treatment, and guide immunosuppressive management.

On the therapeutic front, while antiviral drugs remain central, their limitations, especially in resistant or refractory infections, have driven the development of innovative immunotherapeutic strategies, including adoptive T-cell therapies and notably γδ T-cell-based approaches. These emerging modalities illustrate a paradigm shift from pharmacological suppression of viral replication toward restoration of antiviral immunity capable of achieving durable viral control.

Finally, the meeting emphasized the importance of prevention through optimized vaccination strategies and donor-recipient matching, as well as the necessity of multidisciplinary collaboration in managing complex infectious complications. Despite significant advances, major knowledge gaps remain, particularly regarding rare pathogens, long-term outcomes, and evidence-based management of immunocompromised populations. Continued translational research and well-designed clinical trials are essential to improve the prognosis and quality of care for transplant recipients facing infectious diseases.
